# Long Non-coding RNAs: Emerging Roles in the Immunosuppressive Tumor Microenvironment

**DOI:** 10.3389/fonc.2020.00048

**Published:** 2020-01-31

**Authors:** Ya Luo, Jiqiao Yang, Jing Yu, Xiaowei Liu, Chune Yu, Jianping Hu, Hubing Shi, Xuelei Ma

**Affiliations:** ^1^Laboratory of Tumor Targeted and Immune Therapy, Clinical Research Center for Breast, State Key Laboratory of Biotherapy, West China Hospital, Sichuan University and Collaborative Innovation Center, Chengdu, China; ^2^Department of Breast Surgery, West China Hospital, Sichuan University, Chengdu, China; ^3^College of Pharmacy and Biological Engineering, Sichuan Industrial Institute of Antibiotics, Key Laboratory of Medicinal and Edible Plants Resources Development of Sichuan Education Department, Antibiotics Research and Re-evaluation Key Laboratory of Sichuan Province, Chengdu University, Chengdu, China; ^4^State Key Laboratory of Biotherapy, Department of Biotherapy, Cancer Center, West China Hospital, Sichuan University, Chengdu, China

**Keywords:** immunosuppression, long non-coding RNA, tumor microenvironment, immune response, therapeutic target, biomarker

## Abstract

Tumor immunosuppression may assist the immune escape of cancer cells, which promotes tumor metastasis and resistance to chemo-radiotherapy. The therapeutic strategies against tumor immunosuppression mainly focus on blocking immune checkpoint receptors, enhancing T-cell recognition and neutralizing inhibitory molecules. Although immunotherapies based on these strategies have improved the clinical outcomes, immunological nonresponse and resistance are two barriers to tumor eradication. Therefore, there is an urgent need to identify new biomarkers for patient selection and therapeutic targets for the development of combination regimen with immunotherapy. Recent studies have reported that non-protein-coding modulators exhibit important functions in post-transcriptional gene regulation, which subsequently modulates multiple pathophysiological processes, including neoplastic transformation. Differentiated from microRNAs, long non-coding RNAs (lncRNAs) are reported to be involved in various processes of the immune response in the tumor microenvironment (TME) to promote tumor immunosuppression. Currently, studies on tumor immunity regulated by lncRNAs are mainly confined to certain types of cancer cells or stromal cells. Additionally, the majority of studies are focused on the events involved in T cells and myeloid-derived suppressor cells (MDSCs). Although the reported studies have indicated the significance of lncRNAs in immunotherapy, the lack of comprehensive studies prevents us from exploring useful lncRNAs. In the current review, we have summarized the roles of lncRNAs in tumor immune response, and highlighted major lncRNAs as potential biomarkers or therapeutic targets for clinical application of immunotherapy.

## Introduction

The host immune surveillance evasion by the tumor cells enables them to proliferate, which results in tumorigenesis. The effector T lymphocytes and natural killer (NK) cells have limited efficacy in the tumor microenvironment (TME), due to the gene and/or protein alterations in the tumors and immune cells that are necessary for tumor cell recognition and killing. Additionally, the accumulation of immunosuppressive cells and/or cytokines may suppress the functions of effector immune cells. The TME, which exhibits immunosuppressive characteristics, may affect the outcomes of immunotherapy. Several studies indicated that long non-coding RNAs (lncRNAs) can affect the differentiation and functions of immune cells as well as the progression of tumors. This review summarizes the potential predictive value of lncRNAs for immunotherapy response.

## The Potential Roles of lncRNAs in Cancer Immunotherapy

### The Need for New Biomarkers and Therapeutic Targets for Cancer Immunotherapy

Anticancer immunotherapies, especially immune checkpoint blockade (ICB) and adoptive cellular transfer, are associated with higher efficacy and tolerance than conventional therapies like targeted therapy (1–4). Particularly, the gene signatures indicative of a T cell-inflamed TME in solid tumors are positively correlated with clinical response to anti-programmed cell death protein 1 (PD-1)/programmed death-ligand 1 (PD-L1) therapy (5). Several studies have proposed further stratification of TME to enhance the efficacy of ICBs. Prior to therapy initiation, TME can be classified into four subtypes according to the tumor mutational burden (TMB) and gene signatures for T cell-inflamed TME (5) or can be categorized into three basic immune profiles based on the localization of tumor-infiltrating lymphocytes (TILs) and the expression of PD-L1 in tumor biopsies (6, 7). Although the two stratifications have several differences, they emphasize the immune-inflamed phenotype of the tumors. The inflamed tumor profile is characterized by the infiltration of immune effector cells [e.g., CD8^+^ T cells and type 1 T helper (T_H_1) cells] and proinflammatory cytokines [e.g., interleukin (IL)-12, IL-1β, and type I and type II interferons (IFNs)] in the tumor parenchyma (5, 6). The inflamed tumor samples may be positive for PD-L1 staining. Furthermore, the TILs and immunosuppressive immune cell populations infiltrate inflamed tumors with low TMB. The primary immunosuppressive populations are regulatory T cells (Tregs), myeloid-derived suppressor cells (MDSCs), and tumor-associated macrophages (TAMs). The inflammatory TME may favor tumor growth and metastasis. The inflamed tumor profile, irrespective of the TMB status, indicates that the pre-existing antitumor immune response is markedly suppressed.

The PD-L1/PD-1 blockade deregulates the inhibitory signals transduced into the T cells and subsequently enhances the T cell-mediated cytotoxicity against tumor cells (8). The clinical response to PD-L1/PD-1 blockade ranges from 12 to 52% in the inflamed tumors, such as melanoma, bladder cancer, and mismatch repair-deficient colorectal cancer (9). Some individuals exhibiting high lymphocyte infiltrations and high PD-L1 expression were refractory to anti-PD-L1/PD-1 therapy (5, 10). This indicated that tumor-shrinkage occurs due to a combination of factors and that the immune cell infiltration and PD-L1 expression are necessary but insufficient for tumor response. Hence, several studies are ongoing to identify the biomarkers that can predict the response to PD-1 blockade (pembrolizumab) and to select patients most likely to benefit from immunotherapy. In addition to T cell-inflamed gene expression profile (GEP) and PD-L1 status, TMB and high microsatellite instability (MSI-H) may also act as predictive biomarkers for response to anti-PD-1 therapy (11). Previous studies have reported that TMB and T cell-inflamed GEP can independently predict the response to pembrolizumab. Furthermore, the prediction efficiency of TMB and T cell-inflamed GEP combination was stronger than that of TMB or T cell-inflamed GEP (11). Although TMB and inflammatory biomarkers, including T cell-inflamed GEP and PD-L1 status, have high predictive value for response to pembrolizumab, other hidden biomarkers need to be explored. The mechanisms underlying immunosuppression in inflamed tumors with low TMB are weakly associated with the PD-L1/PD-1 pathway and strongly associated with immunosuppressive immune cell populations. Strategies to relieve immunosuppression mediated by MDSCs, TAMs, and Tregs that activate the effector immune cells may play a major role in tumor regression in these patients. Thus, biomarkers and therapeutic targets focusing on immunosuppressive cells may enhance antitumor immune responses in such tumor types.

### Mechanism of Tumor Immune Escape in T Cell-Inflamed TME

Both tumor-intrinsic and tumor-extrinsic mechanisms are employed by the tumors to evade immune surveillance. The generation of tumor antigens and the expression of major histocompatibility complex (MHC) class I molecules can be decreased to inactivate the T lymphocytes (12). Upon stimulation with IFN-γ, PD-L1 expressed on the tumor cells interacts with PD-1 expressed on the CD8^+^ T cells, which causes T cell exhaustion (13). In addition to the IFN-γ signaling pathway, the loss of sensitivity to tumor necrosis factor (TNF) has been demonstrated to promote tumor immune escape through the upregulation of anti-apoptotic proteins, such as BCL-2. Moreover, the tumor cells can also influence the functions of immune cells via the secretion of immunosuppressive mediators and exosomes that mediate cell-cell communication (13).

During tumor initiation and progression, the TME affects the tissue-resident and blood-derived cells and thus promotes the development of tumors. The following two types of myeloid cells are highly susceptible to environmental signals: dendritic cells (DCs) (antigen presentation) and macrophages (antigen degradation). These cells are influenced by cancer cells through tumor-derived soluble factors, such as IL-6, vascular endothelial growth factor (VEGF), macrophage colony-stimulating factor (M-CSF) (14). Additionally, the production and secretion of IL-1β, granulocyte/macrophage CSF (GM-CSF), prostaglandin E2 (PGE2), and VEGF by tumors promote the expansion of myeloid progenitor cells and immature myeloid cells. This is followed by the accumulation of these cells, which are termed as MDSCs (15). The accumulated DCs, macrophages, and MDSCs facilitate tumor growth by suppressing the proliferation of CD8^+^ T lymphocytes. In the lymphoid compartment, the key effector cells involved in balancing the tumor immunity are CD8^+^ T cells, NK cells, B cells, and CD4^+^ T cells. The CD8^+^ T cells are the predominant anticancer effector cells that give rise to cytotoxic T lymphocytes (CTLs) and kill the tumor cells which present a specific peptide-MHC complex (16). However, the CD8^+^ T cells recruited into the tumor beds also encounter numerous barriers, including the recognition of checkpoints, such as PD-L1/PD-1, CD28/CTLA-4, and immunoglobulin-like transcript receptors (17). Inhibitory molecules, such as T-cell immunoglobulin and mucin-domain containing-3 (TIM-3), lymphocyte activation gene (LAG-3), and T cell immunoreceptor with Ig and ITIM domains (TIGIT) on the Tregs are critical to the suppressive function in antitumor immune response (18). T_H_1-polarized CD4^+^ T cells cooperate with the cytotoxic CD8^+^ T cells to promote the macrophage cytotoxic activities and enhance the antigen presentation by antigen-presenting cells (APCs). Contrastingly, T_H_2-polarized CD4^+^ T cells and other T_H_2 response-initiating cells, such as T_H_2-polarized monocytes and macrophages, and regulatory B cells (Bregs), are crucial pro-tumorigenic components that promote tumor cell survival and proliferation (19, 20). In addition to these cellular components mentioned above, the non-cellular components such as IL-6, IL-10, indoleamine 2,3-deoxygenase (IDO), and TGF-β are also indispensable for the regulation of intratumoral immunosuppression (20, 21).

### LncRNAs as Promising Therapeutic Targets and Biomarkers for Cancer Immunotherapy

LncRNAs, which are the most frequently expressed non-protein-coding transcripts, are localized in the cell nucleus, cytoplasm and exosomes (22) where they interact with various molecules such as DNA, RNA, and proteins. Some lncRNAs that are packaged into the exosomes can function as messengers for signal transduction between the cells (23). Several studies have reported that lncRNAs are involved in pathophysiological processes through the epigenetic, transcriptional, and post-transcriptional regulation of gene expression (24). Additionally, lncRNAs are reported to affect the differentiation and development of myeloid cells and the expression of inflammatory genes in immune cells (22, 25). Furthermore, lncRNAs are also involved in the regulatory circuit of the immune cells. In specific types of immune cells, the expression of lncRNAs is induced by intracellular signaling pathways (such as NF-κB) upon Toll-like receptor (TLR) activation or is suppressed upon cytokine receptor activation, which subsequently regulates the immune responses (25). Previous studies have suggested that some lncRNAs may promote tumor progression through the dysregulation of tumor proliferation, apoptosis (e.g., MA-LINC1, HOTAIR), metastasis (e.g., MALAT1), and angiogenesis (e.g., MIAT, MEG3). LncRNAs target the immune checkpoints and cytokines and promote the formation of the immunosuppressive microenvironment, which contributes to tumor progression and drug resistance. Specifically, lncRNAs are emerging new therapeutic targets and prognostic biomarkers for tissue- and clinical stage-specific cancers (26, 27).

To elucidate the detailed mechanisms underlying the interactions between immune system and tumor cells, researchers are gradually unveiling the roles of lncRNAs in immunosuppressive TME. Computational approaches such as LnCAR (28) and DriverLncNet (29) are developed to explore the functions of lncRNAs in tumor progression based on the causal relations from gene perturbation experiments. In a pan-cancer analysis, the lncRNAs identified by these tools were significantly correlated with the dysregulation of signatures associated with immune responses, including the activation and inhibition of T cells (28, 29). Thus, the strong correlations between lncRNAs and the immune response in cancers warrants further exploration of potential roles of lncRNAs as novel clinical predictors for the efficacy of checkpoint blockades.

## Features and Functional Modules of lncRNAs in Tumor Immune Responses

Non-coding RNAs are RNA molecules that do not code for protein. LncRNAs are defined as non-coding RNAs with at least 200 nucleotides, a length cutoff that distinguishes lncRNAs from small regulatory RNAs, such as piRNA (Piwi-interacting RNAs) and other microRNAs (miRNAs). Genome-wide studies (tilling microarray, RNA sequencing, and chromatin marks) have reveled numerous non-coding transcribed bases in mammals (30). According to the current GENCODE Release (version 31) (https://www.gencodegenes.org/), about 30% of the known genes in human genome are transcribed as lncRNAs. The majority of lncRNAs with low conservation level were considered as transcriptional noises, which are by-products during the transcription and splicing of protein-coding genes. A study based on chromatin immunoprecipitation followed by massively parallel sequencing (ChIP-Seq) revealed several functional lncRNAs (31). The chromatin associated-lncRNAs are usually transcribed by RNA polymerase II and are processed like messenger RNA with additional 5′-capping and 3′-polyadenylated tail (31). The high evolutionary conservation among these lncRNAs indicated that they exhibit biological functions in many pathophysiological activities, such as X-chromosome inactivation (XIST) (32) and imprinting (H19) (33). LncRNAs can be categorized into several types based on the distance between neighboring annotated genes. For example, intronic lncRNAs are transcribed from an intron in the genome, long intergenic lncRNAs (lincRNAs) are transcribed from DNA sequence located between protein-coding genes, and antisense lncRNAs are transcribed from the complementary DNA strand of protein-coding genes (30).

**Figure 1:**
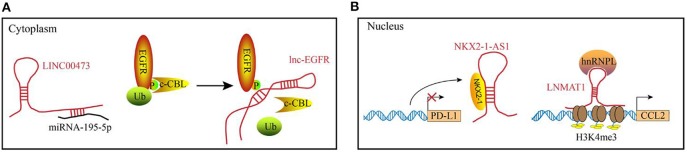
Examples of the functional mechanisms for long non-coding (lncRNAs). LncRNAs function through interacting with diverse molecules in **(A)** the cytoplasm or **(B)** cell nucleus. **(A)** In the cytoplasm, lncRNAs can interact with RNAs or proteins. For example, LINC00473 sponges miRNA-195-5p to reduce its expression level. lnc-EGFR binds to the phosphorylated EGFR to block the ubiquitination, which maintains the activation of EGFR pathway. **(B)** In the nucleus, lncRNA can act as decoys (e.g., NKX2-1-AS1) or guides (e.g., LNMAT1) to regulate the gene expression at transcriptional levels.

The diverse functions of lncRNAs are related to their subcellular localization and targets. In the cytosol, lncRNAs function predominately through RNA-RNA and RNA-protein interactions (Figure 1A). Some lncRNAs act as competing endogenous lncRNAs (ceRNAs) to sponge miRNAs, which results in miRNA dysfunction and subsequently affecting mRNA translation. Recently, lncRNA SNHG1 was reported to directly interact with miR-448 in the regulatory T lymphocytes, which could negatively regulate the expression of IDO (34). In addition to post-transcriptional regulation, several lncRNAs bind to the signaling molecules. For example, Lnc-BM (a lncRNA related to breast cancer brain metastasis), binds to JAK2 and modulates its kinase activity through the Lnc-BM/JAK2/STAT3/ICAM1 pathway (35). Similarly, long intergenic non-coding RNA for kinase activation (LINK-A), interacts with PtdIns(3,4,5) P3 and affects the activation of the AKT pathway in the breast cancer cells (26). LncRNAs play a critical role in the epigenetic and transcriptional regulation of gene expression by interacting with the chromatin (complexes that contain proteins and DNA molecules) within the nucleus. For example, XIST lncRNA acts in cis to silence the gene expression in the X-chromosome of a female by recruiting chromatin modifiers to the adjacent sites (36). Additionally, the DNA-binding domain and specific secondary structures of lncRNAs enable the interaction with one or more proteins and guide them to the specific DNA sites, where they function as decoys, guides and scaffolds to regulate gene expression at the transcriptional level. Examples of the functional modules of lncRNAs in the nucleus are illustrated in Figure 1B.

**Figure 2:**
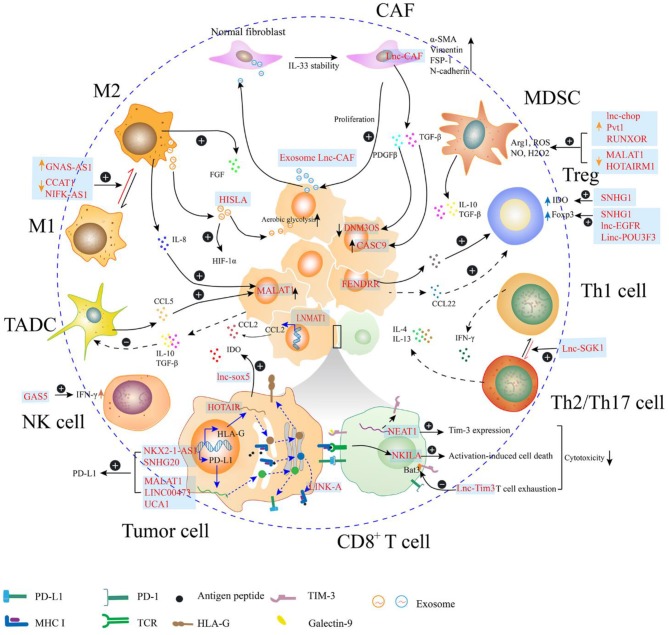
Long non-coding RNAs (lncRNAs) regulate the immunosuppression in the tumor microenvironment (TME). In the TME, lncRNAs regulate the expression of molecules (e.g., PD-L1, MHC I, and HLA-G) on the surface of the tumor cells, which may attenuate the function of effector T cell. Additionally, the cytotoxicity of T cell can be directly regulated by lncRNAs within T cell, through mediating activation-induced cell death or enhancing T cell exhaustion. LncRNAs can also participate in the phenotype transition of cells, such as helper T cell, fibroblast, and macrophage, which can contribute to the formation of immunosuppressive TME. In the myeloid-derived suppressor cell (MDSC), lncRNAs enhance the production of immunosuppressive molecules, such as Arg1 and reactive oxygen species (ROS). Th1, type 1 helper T cell; Th2, type 2 helper T cell; Th17, T helper cell17; Treg, regulatory T cell; MDSC, myeloid-derived suppressor cells; CAF, cancer-related fibroblast; M1, M1 macrophage; M2, M2 macrophage; TADC, tumor-associated dendritic cell; NK cell, natural killer cell.

In the TME, lncRNAs are directly or indirectly involved in the regulation of tumor immunosuppression through multiple mechanisms (Figure 2). Some immunosuppressive cells are generated from their corresponding effector cells through dynamic expression of protein markers in the cell membrane as evidenced by the polarization of macrophages, exhaustion of CD8^+^ T cells, differentiation of helper T cells, and immunosuppressive function of MDSCs. This process can be regulated by endogenous lncRNAs, which regulate gene expression in response to specific stimuli. Moreover, lncRNAs in the tumor cells may facilitate signaling transduction to mediate the degradation of peptide-loading complex (PLC) components, and to sponge miRNAs to upregulate the expression of immune checkpoints, which contribute to decreased immunosurveillance. Furthermore, the lncRNAs encapsulated in the exosomes act as mediators for cell-cell communication in the tumor environment. Exosomes are small membrane-bound vesicles that deliver a specific cargo of proteins and nucleic acids from the parent cells to the recipient cells. Several studies indicate that exosome-transmitted lncRNAs secreted from tumor cells can promote the immunosuppressive function of stromal cells, such as cancer associated fibroblasts and macrophages (37–39). Additionally, the phenomena that exosomes being delivered from immune cells to tumor cells or other kinds of immune cells are observed. On the one hand, TAMs strengthen the aerobic glycolysis and apoptotic resistance of breast cancer cells through the transmission of extracellular vesicles (EVs) with myeloid-specific lncRNA (40). Meanwhile, the exhausted CD8^+^ T cells, which express TIM-3 and PD-1 during the antitumor immune responses, exhibit reduced proliferation and impaired anticancer activities. The regular CD8^+^ T cells uptake exosomes containing lncRNAs secreted by the exhausted CD8^+^ T cells, which leads to CD8^+^ T cell dysfunction (41). Furthermore, some lncRNAs expressed in the tumor cells are affected by immune cells. Tumors are highly organized tissues with numerous reciprocal interactions among distinct cell populations and between cells and soluble molecules. Cytokines, such as CCL5 and IL-8 can be secreted by macrophages and tumor-associated DCs, which interact with the receptors expressed in the tumor cells. The cytokine-mediated interaction enhances cancer progression through lncRNA-dependent pathways, such as the MALAT1/Snail pathway in colon cancer (42).

In the following sections, we will discuss the mechanisms underlying lncRNA-mediated regulation of tumor immune responses. Additionally, we clarify the potential of lncRNAs as biomarkers for patient selection and the possibility to manipulate the expression of lncRNAs for clinical therapeutic applications.

## LncRNAs Modulate Tumor Immunosuppression in TME

LncRNAs are reported not only to mediate crucial signal transductions in cancer signaling pathways but also to affect the tumor immunity to promote tumor evasion from immunosurveillance. Denaro et al. have reviewed that immune cells, such as T cells, B cells, dendritic cells, macrophages, and myeloid cells, regulate cancer immunity through lncRNAs-related pathways. Some lncRNAs have been highlighted as theraputic targets and diagnostic markers in cancers (43). Thus, it is valuable to consider that lncRNAs participating in tumor immunosuppression have the potential for clinical applications. To systematically summarize the research results of lncRNAs regulating tumor immunosuppression, we retrieved literatures in the Pubmed database using the following combination of the search terms: “lncRNA or long noncoding RNA or long non-coding RNA,” “immune suppression or immunosuppressive,” and “tumor microenvironment.” Furthermore, studies on the pivotal immune checkpoints [PD-L1, TIM-3, and human leukocyte antigen (HLA)-G] and immunosuppressive cells (Tregs, MDSCs, and TAMs) that are reported to be regulated by lncRNAs in TME were also included. The studies were excluded based on the following criteria: (a) studies that have reported lncRNAs to regulate the function of immune cells but have not elucidated the roles of lncRNAs in cancers; (b) studies that have reported the role of lncRNAs in tumor metastasis or progression but have not directly demonstrated the interactions between lncRNAs and immune system; (c) studies that have reported the functions of lncRNAs in hematological tumor. The literature analysis indicated that the mechanisms underlying lncRNA-mediated regulation of tumor immunosuppression can be classified according to the type of cells expressing lncRNAs. In the tumor cells, some oncogenic lncRNAs regulate the immunogenicity of tumors by upregulating the expression of immune checkpoints (e.g., PD-L1 and IDO) and HLA-G, or directly by downregulating the generation of tumor antigens. Furthermore, lncRNAs within tumor cells may accumulate in the stromal cells that are recruited into the TME and secrete the suppressive molecules that affect the tumor cell-mediated immunosuppression. The reported studies mainly focused on immunosuppressive and immunoregulatory cells, such as M2 macrophages, MDSCs, and Tregs, which are associated with immunosuppressive TME. LncRNAs that regulate tumor immune escape and the corresponding target genes are described in detail below (Table 1).

**Table 1:** Long non-coding RNAs (lncRNAs) and their respective molecules or pathways involved in the tumor microenvironment (TME) immunosuppression.

**LncRNA**	**Type of cancer**	**Type of cell that express lncRNAs**	**Detected samples**	**Molecules or pathways involved with lncRNA**	**References**
XIST	BCBM	Tumor cell	Tissue, cell line	Exosome miRNA-503	(44)
Lnc-BM	BCBM	Tumor cell	Cell line	JAK2-STAT3-ICAM1, CCL2, IL-6, oncostatin M Lnc-BM/JAK2/STAT3 pathway	(35)
LNMAT1	Bladder cancer	Tumor cell	Tissue, cell line	CCL2, hnRNPL, VEGF-C signaling	(45)
NKILA	Breast cancer Lung cancer	CTL and Th1 cell	Tissue	NF-κB	(46)
SNHG1	Breast cancer	Treg cell	Tissue	miR-448, Foxp3, IDO	(34)
HISLA	Breast cancer	TAM	Cell line	HIF-1α, lactate	(40)
HOTAIR	Cervical cancer	Tumor cell	Tissue, cell line	miR-148a, HLA-G	(47)
CASC9	Cervical cancer	Tumor cell	Tissue, cell line	miR-215/TWIST2 signaling, TGF-β	(48)
GAS5	CRC	Tumor cell	Tissue, serum, cell line	NF-κB, ERK1/2 pathways	(49)
MALAT1	CRC	Tumor cell	Cell line	CCL5, MALAT-1/Snail pathway	(42)
lnc-sox5	CRC	Tumor cell	Tissue, cell line	IDO	(50)
MALAT1	DLBCL	Tumor cell	Tissue, cell line	miR-195, PD-L1	(51)
NIFK-AS1	Endometrial cancer	TAM	Tissue	miR-146a, Notch1	(52)
SNHG20	ESCC	Tumor cell	Tissue, cell line	ATM-JAK-PD-L1 pathway	(53)
DNM3OS	ESCC	Tumor cell	Tissue	PDGFβ-DGFRβ/FOXO1 pathway	(54)
UCA1	Glioblastoma	Tumor cell	Cell line	CXCL14	(55)
CASC2c	GBM	Tumor cell	Tissue, cell line	Coagulation factor X, miR-338-3p, ERK1/2, AKT	(56)
HOTAIR	Gastric cancer	Tumor cell	Tissue	miR-152, HLA-G	(57)
UCA1	Gastric cancer	Tumor cell	Tissue	miR-193a, miR-214, PD-L1	(58)
Lnc-SGK1	Gastric cancer	Infiltrating lymphocyte	Tissue	SGK1	(59)
Linc-POU3F3	Gastric cancer	Treg cell	PBMCs	TGF-β	(60)
FENDRR	HCC	Tumor cell	Tissue	miR-423-5p	(61)
lncTCF7	HCC	Tumor cell	Cell line	IL-6, STAT3	(62)
TUC339	HCC	Tumor cell, macrophage	Cell line	NA	(37)
Lnc-Tim3	HCC	CD8^+^ T cell	Tissue	TIM-3	(63)
lnc-EGFR	HCC	Treg	Tissue	EGFR, Foxp3	(64)
NEAT1	HCC	CD8^+^ T	PBMCs	miR-155, TIM-3	(65)
lincRNA-Cox2	HCC	M1 and M2 macrophage	Macrophages	NA	(66)
GAS5	Liver cancer	NK cell	Tissue	miR-544, RUNX3	(67)
Pvt1	LLC	Granulocytic MDSC	Tissue	NA	(68)
MALAT1	Lung cancer	Tumor cell	Tissue	miR-200a-3p, PD-L1	(69)
NKX2-1-AS1	Lung cancer	Tumor cell	Tissue, cell line	NKX2-1, PD-L1	(70)
MALAT1	Lung cancer	MDSC	PBMCs	NA	(71)
HOTAIRM1	Lung cancer	MDSC	MDSCs and PBMCs	HOXA1	(72)
RUNXOR	Lung cancer	MDSC	Tissue and PBMCs	RUNX1	(73)
AFAP1-AS1	NPC	Infiltrating lymphocyte	Tissue	PD-1	(74)
LIMT	Ovarian cancer	Tumor cell	Cell line	EGF, EGFR-ERK signaling pathway	(75)
Lnc-CAF	OSCC	CAF and tumor cell	CAFs from tissue	IL-33	(38)
LncRNA-MM2P	Osteosarcom	M2 macrophage	Cell line	NA	(76)
LINC00473	Pancreatic cancer	Tumor cell	Tissue, cell line	miRNA-195-5p, PD-L1	(77)
MALAT1	Prostate cancer	Tumor cell	Tissue	IL-8, STAT3	(78)
CCAT1	Prostate cancer	M2 macrophage	Cell line	miR-148a, PKCζ	(79)
MALAT1	Thyroid cancer	TAM	Tissue, cell line	FGF2	(80)
LINK-A	TNBC	Tumor cell	Tissue	PtdIns(3,4,5)P3, GPCR-PKA pathway, TRIM71	(26)
lnc-chop	Variable	MDSC	MDSCs	CHOP, C/EBPβ	(81)

*Variable, 1D8 ovarian cancer, 4T1 breast cancer, B16 melanoma, and Lewis lung cancer cells; CRC, Colorectal cancer; DLBCL, diffuse large B cell lymphoma; ESCC, Esophageal squamous cell carcinoma; TNBC, Triple-negative breast cancer; HCC, hepatocellular carcinoma; GBM, Glioblastoma multiforme; NPC, Nasopharyngeal carcinoma; OSCC, oral squamous cell carcinoma; LLC, Lewis lung carcinoma; BCBM, Breast cancer brain metastasis*.

### Tumor Cell-Derived lncRNAs Mediate Tumor Immunosuppression

#### Expression of Molecules in Tumor Cells

Mutation-derived tumor antigens are expressed on the membrane of cancer cells in the form of peptide-MHC class I complexes, which are recognized by cytotoxic CD8^+^ T lymphocytes that kill the tumor cells. During the antigen presentation of peptide-MHC complexes, the peptides are degraded from the cytosolic proteins by proteasomes. The degraded peptides are then transported into the lumen of the endoplasmic reticulum, where the antigen peptides bind to the MHC class I molecules and are transported to the cell surface (82). The dysregulated antigen presentation machinery in the tumor may facilitate cancer cells to escape the immunosurveillance. The components of PLC play an important role in the transportation and presentation of peptide-MHC complexes. The antigenicity of cancer cells may be reduced through the degradation of PLC components, which are modified by ubiquitin. The expression of LINK-A, a tissue-specific lncRNA, in the triple-negative breast cancer (TNBC) tissues was reported to be higher than that in the non-TNBC tissues. Additionally, LINK-A can predict poor prognosis in patients with breast cancers (83, 84). Hu et al. demonstrated that the expression of LINK-A was negatively associated with the abundance of APCs and CD8^+^ T cells in the basal-like breast cancers, which indicated a correlation between LINK-A and immunosuppression (26). In the transgenic MMTV-Tg (LINK-A) mouse model, LINK-A functions as an oncogenic lncRNA and initiate metastatic mammary gland tumors, which phenotypically resembled the human TNBC. Moreover, LINK-A could enhance the polyubiquitination-mediated degradation of the PLC components and tumor suppressors (Rb and p53) through the inhibitory GPCRs/PKA pathway (26). Treatment with the locked nucleic acids (LNAs) of LINK-A or GPCR antagonists *in vivo* increased the stability of MHC class I complexes and PLC components. Importantly, treatment with LNA did not affect the distribution of immune cells, such as CD8^+^ T cells, macrophages, and MDSCs in the regular mammary glands.

A recent study that tumor cells may upregulate non-classical HLA molecules, such as HLA-G, which can be modulated by cytokines like IL-10 and IFN-γ to evade immunosurveillance. HLA-G binds to the inhibitory receptors expressed on different immune cells, which results in the suppressive immune responses, such as the inhibition of cytotoxicity of CD8^+^ T cells and NK cells (85). Recent studies have reported that HOTAIR, a ceRNA, may modulate the expression of HLA-G by competitively binding to miR-152 (57) or miR-148a (47) in cancer cells. HOTAIR is overexpressed in different types of human malignancies and is involved in cancer progression and metastasis. In patients with cervical cancer, HOTAIR upregulation was correlated with more advanced clinical characteristics and shorter overall survival.

In the T cells, the reduction of tryptophan by indoleamine 2,3-dioxygenase 1 (IDO1) can activate the stress-response kinase GCN2, which inhibits T cell proliferation and induces the differentiation of naïve CD4^+^ T cells into Tregs. Therefore, IDO1 expression in tumors may contribute to immune evasion. Wu et al. reported that lnc-sox5 was upregulated during the tumorigenesis of colorectal cancer (CRC). Additionally, the absence of lnc-sox5 did not affect the growth of tumor cells in immunodeficient mice, but significantly suppressed tumorigenesis in immunocompetent mice (50). Flow cytometry analysis suggested that the knock down of lnc-sox5 promoted the infiltration and the cytotoxicity of CD3^+^CD8^+^ CTLs in tumors in immunocompetent mice. Furthermore, the frequency of Tregs was markedly suppressed. The expression of IDO1 is significantly reduced in Caco-2 cells and MC-38 cells upon lnc-sox5 knockdown. Therefore, lnc-sox5 may serve as a modulator of IDO1 in tumor cells and can be a potential therapeutic target for cancers.

PD-L1 expressed on the tumor cells interacts with PD-1 receptor expressed on the activated T cells, which transduce inhibitory signals for T cell proliferation and cytokine production. LncRNAs are reported to mediate the expression of PD-L1 on tumor cells through various mechanisms. LncRNAs can indirectly upregulate PD-L1 expression by sponging miRNAs. For example, lncRNA UCA1 repressed the expression of miR-193a, miR-26a/b, and miR-214 in gastric cancer through direct interactions and improved the expression of PD-L1 (58). Other studies also reported that lncRNA LINC00473 sponged miR-195-5p to enhance the expression of PD-L1 in prostate cancer (77), while lncRNA MALAT1 regulated tumor migration and immune evasion by modulating the miR-195/PD-L1 axis in diffuse large B-cell lymphoma (51) and the miR-200a-3p/PD-L1 axis in lung cancer (69), respectively. Soluble factors secreted by the immune cells also affect the expression of MALAT1. Kan et al. reported that CCL5 derived from tumor-associated DCs was associated with the up-regulation of MALAT1, which subsequently increased the expression of Snail to promote tumor progression (42). A recent study also reported that IL-8 secreted from M2 macrophages sufficiently promoted the expression level of MALAT1 by activating the STAT3 signaling pathway (78). These studies suggest that MALAT1 serves as a key regulator during tumor progression, especially during tumor immune evasion. LncRNAs can also regulate PD-L1 expression by interacting with proteins (53, 70). NKX2-1-AS1 is an antisense lncRNA that partially overlaps the NKX2-1/TTF1 gene. In lung adenocarcinomas, NKX2-AS1 and NKX2-1 were highly expressed, but NKX2-AS1 did not regulate the expression of NKX2-1 or nearby genes. NKX2-1-AS1 negatively regulated the transcriptional activity of *PD-L1* by interfering with the binding of NKX2-1 protein to the promoter of PD-L1 by potentially functioning as a decoy molecule (70).

#### Pro-tumoral Cytokines

LncRNAs expressed in tumor cells may affect not only the tumor cells but also tumor-directed immune responses. For example, the tumor-suppressive growth arrest-specific transcript 5 (GAS5) lncRNA was reported to be associated with the expression of VEGF-A and IL-10 in the tumor cells (49). VEGF-A is a well-known proangiogenic molecule produced by the tumor cells. Additionally, VEGF-A plays a key role in the induction of immunosuppressive microenvironment by enhancing the expression of inhibitory checkpoint molecules in the CD8^+^ T cells, inhibiting the maturation of DCs, and promoting the differentiation of Tregs (86–88). IL-10 acts as an immunosuppressive cytokine to inhibit the maturation of DCs and the antigen cross-presentation to T cells (89). The tumor tissues exhibit lower GAS5 lncRNA expression than the corresponding normal tissue (90, 91). The knockdown of GAS5 in the CRC cells improved the expression of VEGF-A and IL-10 via the nuclear factor-κB (NF-κB) and Erk1/2 pathways, respectively (49). In the colitis-associated cancer mouse model, GAS5 was markedly down-regulated and was negatively associated with the expression of VEGF-A and IL-10 (49). Thus, VEGF-A and IL-10 cytokines suppressed by GAS5 may serve as targets for lncRNA-based therapeutic regimens against CRC.

#### Effect of lncRNA on Immune Cells Within TME

Several studies suggest that tumor cell-derived lncRNAs may also be involved in the secretion of factors such as CCL2, coagulation factor X (FX), and exosomal miRNAs to promote tumor metastasis by influencing macrophage recruitment and polarization. Chen et al. reported that lymph node metastasis associated transcript 1 (LNMAT1), a novel lncRNA, was significantly upregulated in bladder cancers with lymph node metastasis. Moreover, enhanced LNMAT1 expression was significantly associated with more advanced clinicopathological characteristics, which indicated a poor survival for patients with bladder cancer (45). LNMAT1 is predominantly localized in the nucleus and recruits hnRNPL to the CCL2 promoter, which results in H3K4 tri-methylation and transcriptional activation (45). Subsequently, CCL2 activated by LNMAT1 recruits macrophages into the tumor mass and promotes the lymphatic metastasis via VEGF-C excretion (45). Lnc-BM, a metastasis-related lncRNA, may promote cancer progression in patients with breast cancer brain metastases (BCBMs) by promoting communication between macrophages and breast cancer cells in the brain TME (35). Tumor cell-derived Lnc-BM facilitated STAT3-dependent expression of CCL2 and ICAM1, which mediated macrophage recruitment and vascular co-option in the brain, respectively. The recruited macrophages secreted IL-6 and oncostatin M, which activated the Lnc-BM/JAK2/STAT3 pathway in breast cancer cells (35). Zhang et al. reported that lncRNA CASC2c interacts with miR-338-3p reciprocally to repress its expression, which increases the expression and secretion of FX. FX is involved in the recruitment and M2-polarization of macrophages in glioblastoma (56). LncRNA X-inactive-specific transcript (XIST) functions as a tumor suppressor in brain-metastatic breast cancer (44). XIST was significantly downregulated in brain-metastatic tumors of patients with breast cancer. The knockout of XIST in mammary glands of mice stimulated the growth of the primary tumor and brain metastases. Loss of XIST also enhanced the secretion of exosomal miRNA-503, which triggered the M2-polarization of microglia, and upregulated the immunosuppressive cytokines in microglia and subsequently suppressed T-cell proliferation (44). Tumor-derived exosomal lncRNAs have been indicated as signaling mediators that orchestrate the communications between tumor cells and macrophages in TME. Li et al. demonstrated that hepatocellular carcinoma (HCC) cell-derived exosomes contained overexpressed TUC339 lncRNA, which may be taken up by the non-polarized THP-1 macrophages (37). Additionally, *in vitro* experiments suggested that TUC339 is majorly involved in macrophage polarization (37).

In addition to macrophages, the functions of Tregs and fibroblasts can also be regulated by lncRNAs from tumor cells in TME. The tumor suppressor lncRNA FOXF1 Adjacent Non-Coding Developmental Regulatory RNA (FENDRR) is reported to be a favorable diagnostic biomarker for HCC (92). Microarray analysis suggested that the expression of FENDRR lncRNA in HCC samples was higher than that in the normal samples. Recent studies also suggested that FENDRR lncRNA suppressed the immune escape of HCC cells (61). The HCC cells transfected with siRNA of FENDRR lncRNA exhibited increased expression of TGF-β, IL-10, and VEGF. Furthermore, FENDRR lncRNA could competitively bind to miR-423-5p to upregulate the expression of growth arrest and DNA-damage-inducible beta (GADD45B). Previous studies have indicated that the loss of GADD45B could increase the number of Tregs (93), which may explain the lncRNA FENDRR-mediated inhibition of immune escape that was medicated by Tregs in the HCC cells (61). Ding et al. discovered a tumor cell-derived exosomal lncRNA, namely Lnc-CAF, which was markedly upregulated in the stromal fibroblasts. This novel stromal lncRNA reprogramed normal fibroblasts (NFs) to cancer-related fibroblasts (CAFs) through the Lnc-CAF/IL-33 pathway and promoted the progression of oral squamous cell carcinoma (OSCC) (38). In OSCC patients, the overexpression of Lnc-CAF and IL-33 was positively associated with higher TNM stages at diagnosis, which indicated worse outcomes.

#### Immunosuppressive Cytokines

In TME, the expression of lncRNAs in tumor cells could be modulated by some soluble immunosuppressive cytokines, which promoted tumor immune escape. This regulatory mode suggests a crosslink between stromal cells and cancer cells. In the cervical cancer cells, TGF-β secreted by CAFs can increase the expression of cancer susceptibility candidate 9 (CASC9) lncRNA, which promoted the migration of tumor cells by sponging miR-215 to up-regulate TWIST2 *in vitro* and *in vivo* (48). Furthermore, bioinformatics analysis has predicted the presence of complementary sequences between miR-215 and CASC9. Another study reported that the human esophageal cancer cells and tumor tissues exhibited higher DNM3OS lncRNA than normal samples. The expression of DNM3OS lncRNA can be promoted by CAF-derived PDGF-β in tumor cells through the PDGFβ/PDGFRβ/FOXO1 signaling pathway (54). In glioblastoma, CXCL14 in glioblastoma-associated stromal cells could induce glycolysis and invasion of glioma cells by regulating the UCA1/miR-182/PFKFB2 axis (55). M2-like TAMs may secret EGF to regulate the growth and migration of ovarian cancer cells and the metastasis of ovarian cancer. EGF secreted by M2-like TAMs may inhibit the expression of metastasis-inhibiting LIMT (lncRNA inhibiting metastasis) by activating the EGFR-ERK pathway to stimulate tumor progression in ovarian cancers (75). The proinflammatory cytokine IL-6 in TME may contribute to the development of HCC. IL-6 can activate STAT3, a transcription activator that binds to the promoter regions of lncTCF7, to induce the expression of lncTCF7 in a time- and dose-dependent manner. Additionally, STAT3 knockdown and inhibition of STAT3 activation decreased the expression of lncTCF7 (62).

### Immune Cell-Derived lncRNAs Affect the Immune Responses in TME

Recent studies have reported that, lncRNAs are crucial regulators for the development and functions of several immune cell lineages, which have been elaborately reviewed elsewhere (25, 94). Furthermore, immune cells within the tumor mass usually undergo epigenetic changes to enhance the survival of tumor cells. LncRNAs derived from immune cells in TME are reported to be involved in the expression of membrane molecules and the secretion of cytokines, which are reviewed in detail below.

#### Lymphoid Immune Cells

Recent studies have indicated that lncRNAs may influence the function of tumor-infiltrating T cells. Huang et al. reported that NF-κB-interacting lncRNA (NKILA) sensitized the T cells to activation-induced cell death (AICD), which was exploited by cancer cells to escape immunological destruction (46). Antitumor CTLs and T_H_1 cells were more sensitive to AICD than Tregs and T_H_2 cells in the breast and lung cancer microenvironments. Antigen-stimulated T cells increased the acetylation of histones at the NKILA promoter region, and subsequently enhanced STAT1-mediated transcription of NKILA (46).

In nasopharyngeal carcinoma (NPC), genome expression profiling data of tumor samples suggested that the expression of AFAP1-AS1 lncRNA was significantly associated with that of PD-1. The immunohistochemical analysis revealed that PD-1 and AFAP1-AS1 were co-expressed in the TILs of the NPC tissues. Patients with NPC exhibited poor prognosis when the tumor was positive for both AFAP1-AS1 and PD-1 (74). TIM-3 expressed on the CD8^+^ T lymphocytes is reported to be correlated with the phenotype of immune exhaustion. Lnc-Tim3 lncRNA specifically blocked the interaction between TIM-3 and Bat3 by binding to the intracellular domain of TIM-3, which suppressed the downstream signaling of the Lck/NFAT1/AP-1 pathway (63). *In vitro* experiments indicated that Lnc-Tim3 inhibited the production of IFN-γ and IL-2 in CD8^+^ T lymphocytes, and enhanced the expression of anti-apoptotic genes, such as Bcl-2 and MDM2. Overexpression of Lnc-Tim3 in the Jurkat T cells upregulated the exhaustion-associated markers, such as PRDM1, LAG-3, and PBX3 (63). Thus, Lnc-Tim3 could promote an exhausted-like phenotype in the CD8^+^ T lymphocytes. Yan et al. demonstrated that nuclear-enriched autosomal transcript 1 (NEAT1) lncRNA affected the expression of TIM-3 by modulating the expression of miR-155 (65). NEAT1 lncRNA was highly expressed in the peripheral blood mononuclear cells (PBMCs) and tumor tissues of patients with HCC. Downregulation of NEAT1 lncRNA inhibited the apoptosis of CD8^+^ T cells and enhanced their cytotoxic activity against tumor cells *in vitro*. In the HCC mouse models, the tumor growth was suppressed upon injection with NEAT1-silenced CD8^+^ T cells (65).

The differentiation and distribution of Tregs among PBMCs and tumor tissues of patients may be influenced by lncRNAs. Linc-POU3F3 expressed in Tregs could promote the distribution of Tregs among PBMCs by activating the TGF-β signaling pathway in patients with gastric cancer. *In vitro* co-culture experiments demonstrated that overexpression of linc-POU3F3 in Tregs promoted the proliferation of cancer cells (60). Jiang et al. demonstrated that lnc-EGFR expressed in the Tregs could affect the expression of Foxp3 in HCC cells and maintain the activation of EGFR by preventing the interaction with c-CBL and phosphorylated EGFR, which resulted in the reduction of EGFR ubiquitination (64). Similarly, SNHG1 lncRNA mediates tumor immune escape by affecting the differentiation of Tregs. Additionally, SNHG1 lncRNA regulated the expression of IDO by directly inhibiting the expression of miR-448. SNHG1 silencing in breast tumor cells inhibited tumor growth and downregulated the expression of SNHG1, IL-10, IDO, and Foxp3 (34). The high expression of Lnc-SGK1 lncRNA was correlated with preferable clinical characteristics in patients with gastric cancer having *Helicobacter pylori* (*H. pylori*) infection and high-salt diet. *In vitro* experiments indicated that *H. pylori* infection and high-salt diet could enhance the expression of Lnc-SGK1 by activating the SGK1/JunB signaling pathway, which induces the differentiation of helper T cells toward T_H_2 and T_H_17cells (59).

#### TAMs

Macrophages exhibit a high plasticity, which endows them with diverse functions in response to microenvironment signals (95, 96). The classically activated macrophages (M1) are often generated upon stimulation with pathogen-associated molecular patterns (PAMPs), while the alternatively activated macrophages (M2) are generated upon stimulation with IL-4, IL-13, or IL-10 (97). Oppositely polarized macrophages differ in the expression of receptors, secretion of cytokines and chemokines, and effector functions (97). The M1 macrophages release pro-inflammatory factors, such as IL-6 and TNF-α and contribute to anti-tumoral immune responses (95). The M2 macrophages exhibit pro-tumoral functions by upregulating the expression of IL-10 and immune checkpoint molecules (89). Various strategies have been developed to target the macrophages in tumor mass. For example, therapies that reprogram M2 macrophages to adopt antitumor M1 phenotypes or enhance antigen-presenting capacity are demonstrated to enhance survival in preclinical models (98–100). Recently, several studies have focused on the regulation of macrophage polarization by lncRNAs. In non-small cell lung cancer (NSCLC), GNAS-AS1 lncRNA is highly expressed in the TAMs, tumor cell lines and clinical tumor samples. GNAS-AS1 promoted M2-polarization of macrophages and the progression of NSCLC cells by directly inhibiting miR-4319, which was downregulated by targeting the N-terminal EF-hand calcium binding protein 3 (NECAB3). The GNAS-AS1/miR-4319/NECAB3 pathway promoted tumor progression of NSCLC by shifting the polarization of macrophages (101). The expression of GNAS-AS1 lncRNA was negatively correlated to the overall survival of patients with NSCLC. Similarly, NIFK-AS1 lncRNA served as a ceRNA of miR-146a to modulate M2-polarization of macrophages in endometrial cancer (EC) (52). Additionally, NIFK-AS1 was downregulated in the TAMs. *In vitro* experiments have suggested that NIFK-AS1 overexpression suppresses the M2-polarization induced by IL-4, which further suppresses the proliferation, migration, and invasion of EC cells (52).

LincRNA-Cox2 is a long intergenic ncRNA (lincRNA) located downstream of the mouse Cox2 gene, which mediates the activation and repression of distinct classes of immune genes (102). LincRNA-Cox2 is reported to influence the functions of specific myeloid cell lineages such as macrophages via the TLR-dependent NF-κB pathway (25). Furthermore, Ye et al. reported that M1 and M2 macrophages exhibited differential expression of lincRNA-Cox2 *in vitro* (66). The expression of lincRNA-Cox2 in the M1 macrophages was higher than that in the non-polarized macrophages and M2 macrophages. The lncRNA silencing experiments revealed that the macrophages have a tendency toward an immunosuppressive phenotype, including the overexpression of IL-10 and arginase-1 (ARG-1), and the enhancement of pro-tumoral abilities. Thus, lincRNA-Cox2 may act as a suppressor of M2-polarization of macrophages to inhibit immune evasion and growth of tumor cells (66). Contrastingly, lncRNA-MM2P was reported to be involved in M2-polarization and M2 macrophage-mediated angiogenesis, which may have a potential role in macrophage-mediated tumorigenesis (76). Other lncRNAs, such as MIR155HG and CCAT1 have also been recognized as regulators of macrophage polarization (79, 103).

#### MDSCs

MDSCs are a population of heterogeneous cells comprising myeloid progenitors as well as immature mononuclear and polymorphonuclear cells (104). The chronic inflammation within the TME induced the accumulation of MDSCs, which exhibited immunosuppressive functions via the expression of ARG-1, IL-10, TGF-β, inducible NOS (iNOS), and COX2 (104–106) that promotes tumor growth.

Recent studies have suggested that lncRNAs play important roles in the immunosuppressive functions of MDSCs. The C/EBPβ and C/EBP homologous protein (CHOP) transcription factors are immunosuppressive regulators of MDSCs (107). Gao et al. identified a novel lncRNA, termed lnc-chop in MDSCs that interacts with CHOP and the C/EBPβ isoform, liver-enriched inhibitory protein. This interaction facilitates the activation of C/EBPβ and promotes the expression of immunosuppressive factors, such as NADPH oxidase 2, NO synthase 2, ARG-1, and cyclooxygenase-2 (107). Similarly, RUNXOR lncRNA was highly expressed in MDSCs isolated from tumor tissues of lung cancer. RUNXOR knockdown decreases the expression of immune suppressive molecules, such as arginase-1 in MDSCs (73). Moreover, hypoxia-induced HIF-1α could upregulate the expression of Pvt1 lncRNA in granulocytic MDSCs. Pvt1 lncRNA knockdown markedly impaires the immunosuppressive functions of G-MDSCs *in vitro* and *in vivo* (68). These findings indicate tumor-promoting roles of lncRNAs via regulation of the immunosuppressive function of MDSCs in TME (81).

Additionally, lncRNAs in MDSCs may also act as tumor suppressors. MALAT1 LncRNA is reported to play critical roles in tumorigenesis, angiogenesis, and metastasis. Recently, Zhou et al. reported that the expression of MALAT1 is downregulated in the PBMCs from patients with lung cancer, which was negatively correlated with the percentage of circulating MDSCs (71). *In vitro* experiments indicated that MALAT1 knockdown markedly increased the percentage of MDSCs (71). HOXA transcript antisense RNA myeloid-specific 1 (HOTAIRM1) lncRNA is reported to modulate the differentiation of myeloid cells by targeting HOXA1 (108). The expression of HOTAIRM1 in the PBMCs of patients with lung cancer was lower than that in the PBMCs of healthy controls. Additionally, the expression of HOTAIRM1 and its target gene HOXA1 were negatively associated with the ratio of MDSCs and ARG-1 levels in PBMCs of patients with lung cancer. Furthermore, HOTAIRM1 overexpression could decrease the immunosuppressive functions of MDSCs and suppress the induction of MDSCs from PBMCs of healthy control (72). Therefore, HOTAIRM1/HOXA1 might be a potential therapeutic target for lung cancer (72).

## The Preclinical and Clinical Applications of lncRNAs in Cancer Immunotherapy

Although several studies have demonstrated the role of lncRNAs in the regulation of tumor progression and immune response, there are limited preclinical and clinical application of lncRNAs in cancer immunotherapy. We searched the clinicaltrials.gov website and only found one ongoing observational study on HOTAIR lncRNA as a biomarker for thyroid cancer. This study investigated the ability of HOTAIR in differentiating benign thyroid nodules from malignant nodules (NCT03469544). As reviewed above, HOTAIR upregulates the expression of HLA-G in tumor tissues (47, 57), which indicated the potential role of HOTAIR as a biomarker of tumor immune escape. Other lncRNAs that regulate the pivotal molecules and pathways that are targeted in clinical therapies, such as tumor vaccine, T cell-based therapy, and ICB, can also potentially predict response to tumor immunotherapy.

The efficiency of ICB therapy is essentially dependent on T cell-recognized neoantigens displayed by MHCs on tumor cells. The lack of tumor neoantigen recognition renders tumors insensitive to PD-L1/PD-1 pathway blockade therapy. Thus, neoantigen-based cancer vaccines, including neoantigen vaccine, peptide vaccine, DNA vaccine, RNA vaccine, and DC vaccine, are developed to overcome these limitations in cancer immunotherapy. Some of these vaccines have been investigated in clinical trials (109). In patients with PD-L1^+^ TNBC tissues, the overall response rate to ICB therapy ranges from 10 to 18.5% (110). In an ongoing Phase Ib clinical trial, HLA-A2^+^ Metastatic TNBC is treated using a combination of PVX-410 cancer vaccine and pembrolizumab (NCT03362060). Furthermore, lncRNAs involved in the regulation of antigen presentation may potentially serve as therapeutic targets in tumor vaccines. In the mouse models of breast cancer, LINK-A expression was induced in mammary gland tumors (26). The inhibition of LINK-A expression suppressed the tumor progression. Furthermore, the combinatorial therapy of LINK-A LNAs and ICB synergistically suppressed tumor growth and markedly prolonged the survival of the tumor-bearing mice (26). Therapeutic strategies that restore the tumor antigen presentation pathway may improve the sensitivity to ICB therapy in TNBCs that lack tumor antigenicity. Therefore, LINK-A has great potential as a valid biomarker in predicting the outcomes of patients with TNBC who receive ICB therapy. Additionally, LINK-A can be a potential therapeutic target that sensitizes breast tumors to immune checkpoint inhibitors.

Adoptive T cell therapies, especially chimeric antigen receptor (CAR) T cell therapies, have yielded remarkable clinical efficacies in the treatment of hematological malignancies (111–114). The U.S. Food and Drug Administration (FDA) has approved two CAR T cell products, Yescarta and Kymriah, for treating hematological malignancies (115). However, the efficacy of CAR T cell therapy for solid malignancies has been limited (116, 117). This may be partly due to the lack of CAR T cell trafficking to the tumor site, insufficient activation, and short-term survival of the transferred T cells, and/or the enhanced immunosuppressive TME in tumor mass (115, 117). Various innovative strategies have been explored to overcome these limitations. Here, we highlight the enhancement of the survival of transferred T cells. A recent review has summarized the strategies to improve T cell resistance to apoptosis, such as the use of co-stimulatory domains in CAR genes, the inhibition of specific death receptor pathways, and the insertion of genes for the anti-apoptotic molecules (BCL-XL or BCL-2) (117). For the epigenetic regulation of T cell apoptosis, lncRNAs may act as an adjuvant target of CAR T cell therapy. NKILA is reported to preferentially sensitize antitumor T cells to cell death upon activation by tumor antigens (46). In the mouse model established by Huang et al. CD8^+^ T cells transduced with NKILA shRNA were transferred into immunocompromised mice with established human breast cancer xenografts, which efficiently suppressed the tumor growth. The NF-κB activity, CTL cytotoxicity, and expression of anti-apoptotic genes in the experimental group were enhanced when compared to those in the tumors of the control group (46). NKILA silencing in the transferred TILs and CAR T cells may overcome tumor immune evasion by inhibiting their AICDs, and improve the efficacy of adoptive T cell therapy for cancers.

## Conclusion and Future Perspectives

Recently, the post-transcriptional modulation of gene regulation by lncRNAs was reported to be a key factor in various pathophysiological processes. Analyzing the function and differentiation of immune cells provide us an insight into the functions of lncRNAs in the immunosuppressive TME. In this review, the role of lncRNAs in tumor immune escape or immune surveillance is illustrated. The functions of lncRNAs within tumor cells or immune cells and in mediating the cell-cell communications were discussed. The pivotal roles of lncRNAs in the regulation of immunosuppressive TME implied that lncRNAs can be employed as biomarkers for cancer diagnosis and prognostic evaluation. Additionally, lncRNAs are potential therapeutic targets for cancer.

The expression of some lncRNAs is tissue- or stage-specific. Thus, lncRNA expressions in a certain cancer type could not be validated in other types of cancers. Recently, some studies have reviewed the literatures related to lncRNAs that are associated with various immune cells and stromal cells in adaptive/innate immune system or in TME (22, 23, 25, 43). It is worth noting that most of them have great potential for cancer research in the respect of tumor immunotherapy. The literatures included in this review must be carefully examined. Except for some studies with elaborate design and solid evidence, most conclusions in the literature need to be considered prudently. However, these studies provide us a basis for exploring new biomarkers and therapeutic targets for the clinical application of immunotherapy.

Additionally, further studies are required to explore this novel but far-ranging field. New computational approaches must to be developed to explore the functions of lncRNAs. Moreover, lncRNA-specific animal models should also be established to enable a better understanding of the roles of lncRNAs within TME for clinical application.

## Author Contributions

YL, JYa, XM, and HS conceptualized this review. YL wrote and edited the manuscript. XL, CY, and JH reviewed the manuscript and provided feedback. YL and JYu created the figures. JYa, HS, and XM revised and edited the manuscript.

### Conflict of Interest

The authors declare that the research was conducted in the absence of any commercial or financial relationships that could be construed as a potential conflict of interest.
